# Hereditary prostate cancer in African American families: linkage analysis using markers that map to five candidate susceptibility loci

**DOI:** 10.1038/sj.bjc.6601417

**Published:** 2004-01-20

**Authors:** W M Brown, E M Lange, H Chen, S L Zheng, B Chang, K E Wiley, S D Isaacs, P C Walsh, W B Isaacs, J Xu, K A Cooney

**Affiliations:** 1 Department of Public Health Sciences, Wake Forest University School of Medicine, Winston-Salem, NC 27157, USA; 2 Department of Internal Medicine, University of Michigan Medical School, Ann Arbor, MI 48109, USA; 3 Department of Urology, University of Michigan Medical School, Ann Arbor, MI 48109, USA; 4 Ann Arbor Department of Veteran's Affairs, Ann Arbor, MI 48109, USA; 5 Center for Human Genomics, Wake Forest University School of Medicine, Winston-Salem, NC 27157, USA; 6 Brady Urological Institute, Johns Hopkins Hospital, Baltimore, MD 21287, USA

**Keywords:** prostate cancer, linkage analysis, hereditary cancer syndromes

## Abstract

African American men have the highest incidence of prostate cancer in the world. Despite this statistic, linkage studies designed to localise prostate cancer susceptibility alleles have included primarily men of Caucasian descent. In this report, we performed a linkage analysis using 33 African American prostate cancer families from two independent research groups. In total, 126 individuals (including 89 men with prostate cancer) were genotyped using markers that map to five prostate cancer susceptibility loci, namely *HPC1* at 1q24–25, *PCAP* at 1q42.2–43, *CAPB* at 1p36, *HPC20* on chromosome 20, and *HPCX* at Xq27–28. Multipoint mode-of-inheritance-free linkage analyses were performed using the GENEHUNTER software. Some evidence of prostate cancer was detected to *HPC1* using all families with a maximum NPL *Z* score of 1.12 near marker *D1S413* (*P*=0.13). Increased evidence of linkage was observed in the 24 families with prostate cancer diagnosis prior to age 65 years and in the 20 families with male-to-male transmission. Some evidence of prostate cancer linkage was also detected at markers mapping to *PCAP*, *HPC20*, and *HPCX*. Continued collection and analysis of African American prostate cancer families will lead to an improved understanding of inherited susceptibility in this high-risk group.

Prostate cancer has a higher incidence and mortality among African American men compared to men of all other racial and ethnic groups living in the US (Jemal *et al*, 2002). The explanation for this observation is uncertain and may be due to a combination of dietary, environmental and genetic factors. Family history has been shown to be a strong risk factor for prostate cancer (see review in Bratt, 2002), and this has led many research teams to collect large prostate cancer families with the goal of identifying prostate cancer susceptibility genes using linkage analysis. Review of most published data, however, describes linkage evidence primarily for Caucasian multiplex prostate cancer families identified in North America and/or Europe.

Several population-based studies performed in the US and Canada have demonstrated that the relative risk of prostate cancer attributed to a family history of prostate cancer is similar between individuals of African and Caucasian descent (Hayes *et al*, 1995; Monroe *et al*, 1995; Whittemore *et al*, 1995). For example, Whittemore *et al* (1995) reported data from a population-based case–control study of prostate cancer among individuals of African-, Caucasian-, and Asian descent from Los Angeles, San Francisco, Hawaii, Vancouver, and Toronto. In their study, the odds ratio associated with a family history of prostate cancer was 3.2 for individuals of African American descent (95% confidence interval 2.0–5.0) and 1.9 for individuals of Caucasian descent (95% confidence interval 1.2–1.9). Given the importance of prostate cancer in African American families and the fact that the family history is a significant risk factor for disease in this racial group, we set out to study a set of multiplex prostate cancer families for linkage to five previously reported prostate cancer susceptibility loci: namely *HPC1* at 1q24–25 (MIM 601518) (Smith *et al*, 1996), *PCAP* at 1q42.2–43 (MIM 602759) (Berthon *et al*, 1998), *CAPB* at 1p36 (MIM 603688) (Gibbs *et al*, 1999), *HPC20* on chromosome 20 (Berry *et al*, 2000b), and *HPCX* at Xq27–28 (MIM 300147) (Xu *et al*, 1998).

## MATERIAL AND METHODS

### Patient selection

A total of 33 African American families, including 19 families from the University of Michigan Prostate Cancer Genetics Project (PCGP) and 14 families from the Johns Hopkins University (JHU) Family Collection, were analysed in this report. Each family has a minimum of two genotyped affected men with prostate cancer in a first- and/or second-degree relationship. For the 19 PCGP families, all cases of prostate cancer were confirmed through review of medical records or by independent report of two family members, and all protocols were reviewed and approved by the University of Michigan Institutional Review Board. Subsets of these families have been included in prior linkage reports from our research team (Cooney *et al*, 1997; Lange *et al*, 1999; Bock *et al*, 2001). A total of 14 families were identified at the Brady Urology Institute at JHU primarily through physician referral. The affection status and age at diagnosis were confirmed either through medical records or from two other independent sources. All participants gave full informed consent and protocols were approved by the Johns Hopkins University Institutional Review Board. Subsets of these families have been included in prior linkage reports (Smith *et al*, 1996; Xu *et al*, 1998,^2001^).

### Laboratory methods

Genotyping was performed by either PCR using radiolabelled PCR primers and acrylamide gel electrophoresis (Cooney *et al*, 1997) or using fluorescently labelled makers with PCR products run on ABI DNA sequencers (Smith *et al*, 1996). A common DNA CEPH control (1347-02) was used to standardise allele sizes across experiments. Marker selection and map position for loci on chromosomes 1 and X are provided in Table 1

**Table 1:** Map position of markers used in prostate cancer linkage analysis for the four candidate regions on chromosomes 1 and X

**CAPB**	**Map position (cM)**	**HPC1**	**Map position (cM)**	**PCAP**	**Map position (cM)**	**HPCX**	**Map position (cM)**
*D1S489*	29.9	*D1S452*	188.9	*D1S2678*	256.3	*DXS1205*	87.6
*D1S1597*	29.9	*D1S212*	193.8	*D1S2670*	263.0	*DXS1200*	96.4
*D1S402*	31.0	*D1S466*	198.3	*D1S2785*	266.3	*DXS1193*	97.9
*D1S407*	33.8	*D1S158*	200.6	*D1S321*	267.5	*DXS1113*	98.1
*D1S3669*	37.1	*D1S422*	205.4	*D1S304*	267.5	*DXS1108*	104.3
*D1S552*	45.3	*D1S413*	212.4	*D1S2842*	273.5		

**Figure 1:**
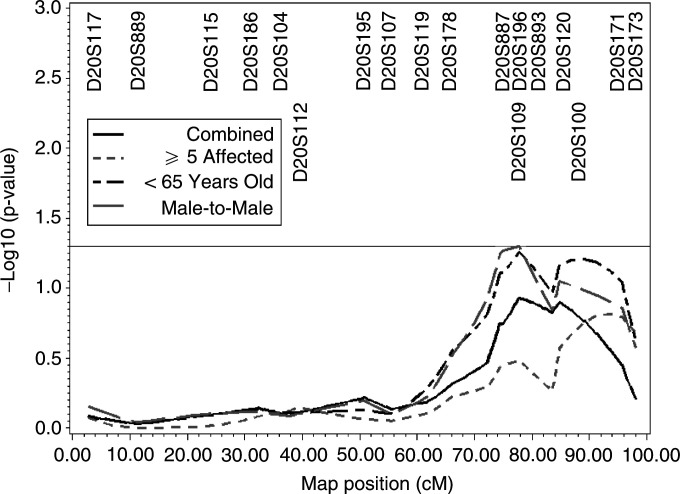
Linkage analysis using markers that span chromosome 20. Markers used for this analysis are illustrated across the top of the figure, and their map position is depicted across the bottom. For all 27 families as well as each stratification, −log 10 of the *P*-value is plotted on the *y*-axis. Only the stratifications of ⩾5 affecteds, age at diagnosis <65 years, and male-to-male transmission are shown.

. The panel of *HPC20* markers used in the current study was nearly identical to those reported in Berry *et al* (Berry *et al*, 2000b) and are shown in Figure 1

. Marker positions were determined by the Mammalian Genotyping Service (MGS) website (http://research.marshfieldclin ic.org/genetics/Map_Markers/ma ps/IndexMapFrames.html).

### Statistical analyses

Multipoint mode-of-inheritance-free linkage analyses were performed using the statistical software package GENEHUNTER versions 1.3. (for chromosome X) and 2.0*β* (for chromosomes 1 and 20) (Kruglyak *et al*, 1996). The marker allele frequencies were estimated from the data using all genotyped individuals. Results are reported in terms of standardised *Z* scores and their associated *P*-values. In addition to analyses of the complete set of pedigrees, stratified analyses were also performed on the subsets of pedigrees using previously defined cutoff values for average age-of-diagnosis of genotyped affected individuals, number of confirmed affected individuals, and classification of male-to-male disease transmission.

## RESULTS

A total of 126 individuals (including 89 confirmed prostate cancer cases) were genotyped and included in the analyses. The clinical characteristics of the families are described in Table 2

**Table 2:** Characteristics of African American prostate cancer families

	**No. of families**	**Ave. no. of confirmed affected men/family**	**Ave. no. of confirmed affected men genotyped/family**	**Ave. age at diagnosis of cases/family^a^**	**No. Families with ave. age at diagnosis 65 years^a^**	**No. families with male-to-male transmission**
University of Michigan PCGP	19	4.4±2.4	2.5±0.7	60.8±6.6	13	12
JHU family collection	14	5.4±3.5	3.1±1.5	59.5±6.8	11	8

Based on those men available for genotyping.

. Seven of 33 families had very early-onset prostate cancer with an average age of prostate cancer diagnosis within the family (based on ages of men available for genotyping) of <55 years. Note that 20 out of 33 or 61% of families had evidence of disease transmission from father to son that would be inconsistent with prostate cancer risk being transmitted from the X chromosome.

Three regions of chromosome 1 were assessed for prostate cancer linkage using a panel of polymorphic markers within each region corresponding to the *CAPB*, *HPC1*, and *PCAP* putative susceptibility genes. Some evidence in support of prostate cancer linkage was observed in the *HPC1* candidate region with a maximum multipoint NPL *Z* score of 1.12 (*P*=0.13) near marker *D1S413* (Table 3

**Table 3:** Summary of mode-of-inheritance-free linkage analyses using markers that map to the three candidate loci on chromosome 1

				
**Chromosome 1Families**	**No. of families**	**Max NPL**	***P*-value**	**Nearestmarker**
**Region 1: CAPB**				
All	32	−0.20	0.58	D1S552
=> 5 affected	13	−0.28	0.59	*D1S552*
< 5 affected	19	−0.03	0.51	*D1S552*
=> 65 years	8	−0.01	0.52	*D1S552*
< 65 years	24	−0.23	0.59	*D1S552*
Male-to-male	20	−0.23	0.58	*D1S552*
Non male-to-male	12	−0.04	0.51	*D1S552*
				
**Region 2: HPC1**				
All	32	1.12	0.13	D1S413
=> 5 affected	13	0.75	0.22	*D1S413*
< 5 affected	19	0.91	0.18	*D1S158*
=> 65 years	8	−0.03	0.52	*D1S552/D1S452*
**< 65 years**	**24**	**1.68**	**0.05**	***D1S413***
**Male-to-male**	**20**	**1.73**	**0.04**	***D1S413***
Non male-to-male	12	−0.02	0.50	*D1S552/D1S452*
				
**Region 3: PCAP**				
All	32	0.59	0.27	*D1S2785*
=> 5 affected	13	0.37	0.34	*D1S413/D1S2678*
< 5 affected	19	0.88	0.18	*D1S2785*
=> 65 years	8	−0.67	0.73	*D1S413/D1S2678*
< 65 years	24	1.48	0.07	*D1S2785*
Male-to-male	20	0.88	0.18	*D1S413/D1S2678*
Non male-to-male	12	−0.07	0.52	*D1S321*

Data from the marker with the most positive NPL *Z* score are provided. Rows that are in bold text indicate NPL *Z* scores that are associated with statistically significant *P*-values (*P*<0.05). One PCGP family was not genotyped for chromosome 1 markers.

). The evidence for linkage was greater in the 24 families with an average age of prostate cancer diagnosis <65 years (*Z*=1.68 near marker *D1S413*; *P*=0.05) and the 20 families with male-to-male transmission (*Z*=1.73 near marker *D1S413; P*=0.04). The highest NPL *Z* score for the *PCAP* locus was observed in the subset of 24 families with an average age of prostate cancer diagnosis <65 years (maximum multipoint NPL *Z* score of 1.48 near *D1S2785*, *P*=0.07). The multipoint NPL *Z* scores for markers corresponding to the *CAPB* locus were largely negative.

A panel of chromosome 20 markers spanning nearly the entire chromosome was used to assess the degree of linkage to *HPC20* given the less exact location for this prostate cancer susceptibility locus (Figure 1). Genotype data was available for 27 of the 33 families. The maximum multipoint NPL *Z* score for the entire set of African American prostate cancer families was 1.17 near marker *D20S893* (*P*=0.12) (Table 4

**Table 4:** Summary of mode-of-inheritance-free linkage analyses using markers that map to the HPC20 and HPCX candidate regions

	**No. of families**	**Max NPL**	***P*-value**	**Nearest marker**
**Chromosome 20 families**				
All	27	1.17	0.12	D20S893
=> 5 affected	11	1.02	0.15	*D20S171*
< 5 affected	16	1.46	0.07	*D20S120*
=> 65 years	7	0.58	0.28	*D20S195*
**< 65 years**	**20**	**1.60**	**0.05**	**D20S893**
**Male-to-Male**	**17**	**1.64**	**0.05**	***D20S893***
Non male-to-male	10	0.33	0.36	*D20S186*
				
***Chromosome X families***				
All	30	1.20	0.12	*DXS1205*
=> 5 affected	12	0.77	0.21	*DXS1200*
< 5 affected	18	1.09	0.15	*DXS1205*
=> 65 years	8	0.83	0.17	*DXS1205*
< 65 years	22	0.77	0.19	*DXS1200*
Male-to-male	18	0.84	0.22	*DXS1205*
Non male-to-male	12	0.86	0.20	*DXS1205*

Data from the marker with the most positive NPL Z score is provided. Rows that are in bold text indicate NPL *Z* scores that are associated with statistically significant *P*-values (*P*< 0.05). Note that six PCGP families were not genotyped for chromosome 20 markers and one PCGP family was not genotyped for chromosome X markers.

). However, as with *HPC1*, increased evidence of prostate cancer linkage was observed in the families with male-to-male transmission (*Z*=1.64; *P*=0.05) and with average age of prostate cancer diagnosis less than age 65 years (*Z*=1.60; *P*=0.05) near the same marker. Increased evidence of linkage was also detected in the families with fewer than five affected family members near marker *D20S120* (*Z*=1.46, *P*=0.07).

The overall evidence of prostate cancer linkage to *HPCX* was weakly positive with a maximum NPL *Z* score of 1.20 near marker *DXS1205* (*P*=0.12) in 30 families. Note that two additional families were removed from this analysis because all affected individuals in these pedigrees could not share the same X chromosome (e.g. paternal uncle and nephew pairs). None of the stratifications provided increased evidence in support of *HPCX* compared to the entire set of families.

## DISCUSSION

This is the first report of prostate cancer linkage to putative prostate cancer susceptibility loci using only families who are of African American descent. Previous studies, including several from our own research groups, have addressed potential prostate cancer susceptibility loci in African American families through stratification of large family data sets according to race. In this report, we have combined two sets of African American pedigrees from the University of Michigan PCGP and the JHU Family Collection. While a subset of these pedigrees have been studied previously, reanalysing the data in a combined analysis facilitates stronger conclusions regarding evidence for linkage to the described candidate regions in African Americans. The number of pedigrees has been expanded since the original reports, and we have attempted to use common markers for each locus. Furthermore, the increased number of pedigrees enables subset analyses incorporating only African American pedigrees. Subsetting pedigrees based on numbers of confirmed affected men, age at diagnosis, and evidence of male-to-male transmission has been invaluable in helping to localise candidate regions for prostate cancer susceptibility genes. Small individual collections of African American pedigrees have, until now, made the approach of stratified analyses infeasible. The increased number of pedigrees also enables better estimates of allele frequencies in the African American population to be used in these analyses. This is relevant since allele frequencies may vary according to race, and mis-specification of allele frequencies may lead to spurious linkage results. Finally, trying to piece together results from nonparametric linkage analysis across different studies to assess the total evidence for linkage is problematic. NPL *Z* scores are nonadditive, and thus the evidence for linkage cannot be simply summed up over all studies. While cumulative *Z* scores may be determined if the number of pedigrees in each study is known, calculating the degree of statistical significance (*P*-value) is far more difficult. Taken together, there is a definite benefit to examining sets of pedigrees together as demonstrated by the analyses presented in this report.

*HPC1* at 1q24–25 has been the most widely studied prostate cancer susceptibility locus. Although there have been both positive and negative linkage reports, a meta-analysis using 772 hereditary prostate cancer families from throughout the world revealed some evidence for prostate cancer linkage with a peak NPL *Z* score of 1.14 at *D1S212* (Xu, 2000). In this meta-analysis, the 491 families with evidence of male-to-male disease transmission revealed increased evidence for linkage with a peak NPL score of 2.30 at *D1S452*. Review of earlier *HPC1* linkage reports suggests that African American prostate cancer families may have evidence of disease linkage to 1q24–25 markers. In the Smith *et al* (1996) study, the two African American families studied had a combined LOD score of 1.4. This was followed up in a report from the University of Michigan PCGP describing disproportionate evidence for 1q24–25 linkage in six African American families from the total sample of 59 families (Cooney *et al*, 1997). Our current combined analysis is consistent with these earlier reports with evidence for linkage to *HPC1* in 32 families (for which genotype data was available) studied together, as well as increased evidence for linkage in families with early-onset disease and in families with male-to-male transmission. Previous work from Gronberg *et al* (1997) has emphasised that young age at diagnosis may be a feature of *HPC1*-related prostate cancers. Recently, *RNASEL* has been proposed to be a candidate for *HPC1* (Carpten *et al*, 2002), and a single African American prostate cancer family has been shown to have a mutation in the initiating methionine occurring in four of six brothers with prostate cancer. Sequencing of 16 African American probands from a set of University of Michigan PCGP families failed to identify this M1I or any other *RNASEL* mutations (Chen *et al*, 2002), Further studies should be performed to specifically test the role of *RNASEL* mutations in African American prostate cancer families.

The two other prostate cancer susceptibility loci on chromosome 1 have not been specifically studied in African American prostate cancer families, although the 14 JHU families were included in a previously published analysis of all three chromosome 1 loci (Xu *et al*, 2001). The *CAPB* locus at 1p36 was described by Gibbs *et al* (1999) in families with an excess of prostate cancer as well as one or more cases of brain cancer in a first- and/or second-degree relative. Review of the pedigrees from the 33 African American families described in this report reveals no cases of primary brain cancer in first- and/or second-degree relatives. Most of the linkage evidence in support of the *PCAP* locus at 1q42–43 is derived from families from France and England (Berthon *et al*, 1998; Cancel-Tassin *et al*, 2001); many of the published prostate cancer linkage studies in other populations have not provided significant support for this putative locus (for example, Berry *et al*, 2000a; Xu *et al*, 2001). In our study of 32 African American families in which genotype data was available, we did not see significant evidence for linkage. However, the 24 families with average age of prostate cancer diagnosis less than age 65 years revealed some evidence for linkage with an NPL *Z* score of 1.48 near marker *D1S2785* (*P*=0.07). This is the same strata that contributed significantly to the observation of prostate cancer linkage in families from Southern and Western Europe (Cancel-Tassin *et al*, 2001). Given the likelihood of locus heterogeneity for prostate cancer, stratification by clinical phenotype (including other cancers segregating in families and young age at diagnosis) may help to elucidate the prostate cancer susceptibility genes relevant to specific types of prostate cancer.

The *HPCX* locus was first described by Xu *et al* (1998) using a set of 360 prostate cancer families. This locus is 50 cM distal to the androgen receptor locus, which maps to Xq11–q12. A previous report from the University of Michigan PCGP provided some additional support of the existence for the *HPCX* locus with positive NPL *Z* scores across the candidate region. However, 11 African American families demonstrated no evidence of *HPCX* linkage with negative NPL *Z* scores across the entire candidate region (Lange *et al*, 1999). Addition of 19 more families in this report has resulted in positive, although not statistically significant, NPL *Z* scores using these same markers. Unfortunately, some of the families described here exhibit only male-to-male transmission of disease. Thus, the stratifications result in small sets of families that limit the conclusions that can be drawn from this analysis.

Regarding the *HPC20* locus, 14 African American prostate cancer families from the PCGP have previously been shown to provide evidence for prostate cancer linkage to this locus with a *Z*_lr_ of 1.99 between markers *D20S893* and *D20S120* (*P*=0.023) (Bock *et al*, 2001), whereas the 14 JHU families revealed negative NPL *Z* scores using a similar set of markers. Despite these conflicting results, the combined analysis of the 27 African American families reported herein reveals suggestive evidence of linkage to *HPC20* with a peak multipoint NPL *Z* score of 1.04 near *D20S893* (*P*=0.15). The subsets of African American families providing the greatest evidence for linkage to *HPC20* contains families with evidence of male-to-male transmission (*Z*=1.64, *P*=0.05) and the families with average of prostate cancer diagnosis <65 years (*Z*=1.60, *P*=0.05). This contrasts with the initial report describing *HPC20* in which the subset with the strongest evidence of linkage to chromosome 20 markers was the 19 white families with <5 affected family members, average age of prostate cancer diagnosis >66 years and no evidence of male-to-male transmission (Berry *et al*, 2000b).

It should be noted that two African American JHU families included in this report were also included in the original study by Smith et al. (Smith *et al*, 1996), which suggested the importance of *HPC1* and *HPCX* in prostate cancer susceptibility. Removing these two families from this current analysis resulted in slightly reduced statistical significance estimates within these regions. For example, the statistical significance of the evidence for linkage at marker *D1S413* in families with male-to-male transmission changed very modestly (from *P*=0.04 to *P*=0.07). Given the limited number of currently available African American pedigrees, we have chosen to include these two families in the analyses of *HPC1* and *HPCX* described in this report.

In conclusion, this combined analysis of 33 African American prostate cancer families provides continued support for the *HPC1* and *HPC20* loci as potentially important contributors to prostate cancer susceptibility in this population. Analysis of these additional African American prostate cancer families now provides some evidence that *HPCX* may contribute to prostate cancer susceptibility in this racial subgroup. Additionally, some new evidence for the *PCAP* locus was identified particularly among families with early-onset prostate cancer. Clearly, given the still relatively small sample size of the current study and the resulting modest power to detect linkage when present, no firm conclusions should be drawn regarding the other candidate regions in regards to susceptibility in the African American population. Continued support for collection of DNA samples from African American prostate cancer families and genome-wide analyses should help illuminate the predisposing genes in this high-risk population. However, given the relative difficulty in ascertaining African American pedigrees, it is imperative that additional cooperative studies such as this be undertaken to increase our ability to localise and assess the role of prostate cancer susceptibility genes in this population.
